# Genome-wide identification and functional characterization of *LEA* genes during seed development process in linseed flax (*Linum usitatissimum* L.)

**DOI:** 10.1186/s12870-021-02972-0

**Published:** 2021-04-21

**Authors:** Zhen Li, Hui Chi, Caiyue Liu, Tianbao Zhang, Lida Han, Liang Li, Xinwu Pei, Yan Long

**Affiliations:** Biotechnology Research Institute, Chinese Academy of Agricultural Sciences, Beijing, 100081 China

**Keywords:** *LEA* gene identification, Functional analysis, Seed development, Fatty acid, Linseed flax

## Abstract

**Background:**

LEA proteins are widely distributed in the plant and animal kingdoms, as well as in micro-organisms. *LEA* genes make up a large family and function in plant protection against a variety of adverse conditions.

**Results:**

Bioinformatics approaches were adopted to identify *LEA* genes in the flax genome. In total, we found 50 *LEA* genes in the genome. We also conducted analyses of the physicochemical parameters and subcellular location of the genes and generated a phylogenetic tree. *LuLEA* genes were unevenly mapped among 15 flax chromosomes and 90% of the genes had less than two introns. Expression profiles of *LuLEA* showed that most *LuLEA* genes were expressed at a late stage of seed development. Functionally, the *LuLEA1* gene reduced seed size and fatty acid contents in *LuLEA1*-overexpressed transgenic *Arabidopsis* lines.

**Conclusion:**

Our study adds valuable knowledge about *LEA* genes in flax which can be used to improve related genes of seed development.

**Supplementary Information:**

The online version contains supplementary material available at 10.1186/s12870-021-02972-0.

## Background

Late embryogenesis abundant (LEA) proteins are widespread in multiple types of tissues of living organisms [1, 2]. These proteins have been observed in bacteria, cyanobacteria [3], fungi and animals [1, 3] but were first discovered in mature cotton seed by researchers in 1981 [4]. As the name implies, this protein accumulates during the late stage of seed maturation. Subsequent discoveries identified the protein in other plants, such as rice, *Arabidopsis thaliana*, maize [1, 5, 6], etc. [7–9]. In plants, *LEA* genes express in many different tissues, such as seeds, roots, stems, and buds [10], so their potential functions are not limited to the process of seed development. Scientists have identified that LEA proteins can be induced to express and function as protectants of proteins and membranes in unique ways when cells are under stress, in particular drought and desiccation. Most LEA proteins are low-weight molecules ranging in size from 10 to 30 kD.

Several classifications of LEA proteins have been identified according to different standards. A widely adopted classification sorts the LEA proteins into eight subgroups: LEA_1, LEA_2, LEA_3, LEA_4, LEA_5, LEA_6, dehydrin and seed maturation protein (SMP). This classification is based on the sequence homology and conserved motifs available in the Pfam database [2, 5]. Among the eight LEA subgroups, with the exception of a few atypical hydrophobic proteins in the LEA_2, LEA_3 and SMP subgroups [11], the proteins possess high contents of Arg/Lys, Glu, Ala, Thr and Gly [12]. All dehydrin proteins have K-segments that are rich with lysine, and some even have Y-segments or S-segments. These segments can exist in the form of tandem repeats [13]. Unlike other proteins, most LEA proteins that possess intrinsically disordered proteins (IDPs) have no three-dimensional structures [14, 15], which accords with their high hydrophilicity.

Seed development, a crucial part of the angiosperm life cycle, is regulated by a large intricate network involving multiple factors, including transcription, epigenes, hormones, peptides and sugar signaling regulators [16]. In general, seed development can be roughly divided into two phases, morphogenesis and maturation [17]. Of the latter phase, strong expression of LEA proteins is regarded as a clear indication of seed maturation [18, 19]. Previous studies indicate that LEA proteins might be related to seed longevity, desiccation tolerance, and viability [20–23]. A subset of LEA proteins are regulated by a network of transcription factors containing ABI3, ABI4, ABI5, EEL and DOG1, as evidenced by the down-regulation of LEA transcripts in *abi3, abi5, leafy cotyledon1* and *fusca3* mutants [18, 24]. The transcription factors LEC1, FUSCA3, and ABI3 are involved in fatty acid biosynthesis and lipid storage in seeds [25]. However, little evidence demonstrates that LEA proteins control seed traits directly or indirectly. This may be because most research has been focused on the contributions of LEA proteins to the tolerance of drought, heat, cold and other abiotic stresses [19, 26]. To our knowledge, only Liang et al. (2019) demonstrated that overexpression of *LEA3* in *Arabidopsis* and *Brassica napus* enhanced seed, seed weight, and oil content [27]. Overall, our knowledge on how LEA proteins are involved in seed development and the lipid-regulated network still have many gaps to fill. Moreover, LEA proteins in every subfamily exhibit different functions, thus these potential functions are additional gaps of knowledge that need to be filled.

With the development of rapid sequencing technology, more and more plant genomic information has become available. In the last 20 years, many LEA proteins have been identified in different plant species, including rice [6], *A. thaliana* [5], maize [28], *B. napus* [29], sorghum [30], watermelon [7], and wheat (*Triticum aestivum*) [31]. Additionally, studies report most LEA proteins in plant species have many members, for example, the numbers of members are 51 in *A, thaliana* [5], 108 in *B. napus* [29], and 281 in wheat [32]; the relatively high numbers reflects their significant role in plants. However, still unknown are the precise functions of most LEA genes.

Flax (*Linum usitatissimum* L.), a self-pollinating annual herb, has a long history of domestication of 8000 years, originated in the Middle East, and now is widely distributed around the world [33]. Flax is classified into two types, fiber flax and linseed flax, based on how each are utilized. Current linseed flax varieties are able to accumulate up to 50% oil content in seeds, and the majority of the fatty acids are composed of palmitic acid (PAL; C16:0, ~ 6%), stearic acid (STE; C18:0, ~ 2.5%), oleic acid (OLE; C18:1,~ 19%), linoleic acid (LIO; C18:2, ~ 13%) and linolenic acid (LIN; C18:3, ~ 55%) [34]. Distinct from most oil-bearing crops, linseed contains a diversity of amino acids and vitamins and a much higher level of unsaturated fatty acids, in particular alpha-linolenic acid (ALA), which accounts for up to 64% of unsaturated fatty acids in flax seed oil [35]. The fatty acid ALA and its transformations such as DHA (docosahexaenioc acid), EPA (eicosapntemacnioc acid) are greatly benefited for people health care.

Because the genome sequence of flax is available for study [36], researchers can more easily identify *LEA* genes in flax. In this study, several *LuLEA* genes were identified in the flax genome. Gene structure and phylogenic analyses showed that the genes could be classified into eight subgroups. Additionally, we determined gene expression levels during the seed development process. Lastly, from among the *LuLEA* genes that expressed abundantly at the late maturation stages, we selected one *LEA* gene, *LuLEA1*, to transform into *Arabidopsis*. The *LuLEA1*-over-expression lines produced seeds reduced in size and fatty acid contents compared to those in the WT (wild type). Our results will not only help improve understanding of the LEA family in the flax genome, but also provide insights into LEA functions correlating with oil metabolism in flax.

## Results

### Identification of *LuLEA* gene families in the flax genome

Combining the methods of local BLAST with HMM, 50 *LuLEA* gene members of the *LEA* family were identified the flax genome (Table 1). These genes were named in order from *LuLEA1* to *LuLEA50*. Based on the sequence homology and conserved motifs in the Pfam database, these *LuLEA* genes were divided into eight subfamilies, the LuLEA_1, LuLEA_2, LuLEA_3, LuLEA_4, LuLEA_5, LuLEA_6, dehydrin, and SMP subfamilies. Among the subfamilies, the dehydrin subfamily had the highest number of genes, 10. Following the dehydrin group were the LuLEA_1, LuLEA_2, LuLEA_3 subfamilies with 9, 8, and 8 genes respectively. The smallest subfamilies were LuLEA_4 and LuLEA_6 in which each had two gene members.

**Table 1:** *LEA* genes in the linseed flax genome and their sequence characteristics and physicochemical parameters

Code	gene ID	subfamily	chromosome	start position	end position	gene length	amino acid number	molecular weight	pI	GRAVY (Grand average of hydropathicity)
LuLEA1	Lus10016273	LEA_1	1	8,834,131	8,834,846	716	171	17,497.09	8.02	−0.878
LuLEA2	Lus10016266	LEA_1	1	8,852,939	8,853,333	395	95	9972.04	9.26	−0.971
LuLEA3	Lus10004182	LEA_1	6	15,603,142	15,603,898	757	192	19,522.54	8.07	−0.675
LuLEA4	Lus10012018	LEA_1	1	5,275,588	5,276,273	686	163	16,805.51	8.83	−0.868
LuLEA5	Lus10012009	LEA_1	1	5,291,874	5,292,273	400	94	9955.05	9.26	−0.989
LuLEA6	Lus10030959	LEA_1	9	5,737,361	5,737,983	623	180	19,210.83	9.59	−0.326
LuLEA7	Lus10043356	LEA_1	12	18,006,089	18,006,485	397	101	10,984.61	9.64	−0.974
LuLEA8	Lus10040088	LEA_1	7	4,983,805	4,984,203	399	105	11,585.3	10	−0.585
LuLEA9	Lus10021044	LEA_1	8	5,446,756	5,447,674	919	275	28,147.83	9.25	−0.785
LuLEA10	Lus10007905	LEA_2	15	15,218,505	15,218,906	402	133	14,348.51	5.03	0.067
LuLEA11	Lus10010140	LEA_2	1	27,748,453	27,748,974	522	173	18,824.58	6.91	−0.191
LuLEA12	Lus10010139	LEA_2	1	27,744,603	27,745,160	558	158	17,017.53	4.84	0.089
LuLEA13	Lus10019367	LEA_2	3	9,280,916	9,281,338	423	140	15,636.12	5.37	0.039
LuLEA14	Lus10036402	LEA_2	11	7,987,612	7,988,013	402	133	14,338.52	5.03	0.063
LuLEA15	Lus10008337	LEA_2	14	10,638,391	10,639,780	1390	360	40,100.87	4.82	−0.255
LuLEA16	Lus10001869	LEA_2	1	27,744,604	27,745,160	557	158	17,087.67	4.97	0.098
LuLEA17	Lus10001876	LEA_2	scaffold78	62,191	76,313	14,123	449	49,626.94	5.26	−0.381
LuLEA18	Lus10006508	LEA_3	12	6,574,065	6,574,454	390	93	9671.89	8.04	−0.315
LuLEA19	Lus10029634	LEA_3	9	18,092,448	18,092,717	270	89	9562.94	9.99	−0.288
LuLEA20	Lus10037497	LEA_3	3	25,716,318	25,716,712	395	109	11,650.1	9.25	−0.472
LuLEA21	Lus10008169	LEA_3	14	10,147,208	10,147,453	246	81	8669.03	10	−0.249
LuLEA22	Lus10008170	LEA_3	14	10,143,342	10,143,614	273	90	9411.64	9.99	−0.301
LuLEA23	Lus10027986	LEA_3	1	25,394,304	25,394,585	282	93	9850.21	9.8	−0.171
LuLEA24	Lus10027987	LEA_3	1	25,391,291	25,391,542	252	83	8817.13	10.08	−0.293
LuLEA25	Lus10042672	LEA_3	10	13,440,188	13,440,457	270	89	9725.22	10.13	−0.345
LuLEA26	Lus10035586	LEA_4	1	8,052,046	8,053,330	1285	398	43,529.02	5.56	−1.120
LuLEA27	Lus10008638	LEA_4	1	4,084,499	4,086,081	1583	497	54,396.05	5.57	−1.166
LuLEA28	Lus10005044	LEA_5	1	8,624,064	8,624,477	414	113	12,262.34	5.78	−1.393
LuLEA29	Lus10030394	LEA_5	4	1,363,184	1,363,542	359	92	9929.79	6.61	−1.424
LuLEA30	Lus10027816	LEA_5	1	4,694,779	4,695,196	418	113	12,262.34	5.78	−1.393
LuLEA31	Lus10037844	LEA_5	15	14,416,820	14,417,098	279	92	9907.74	5.59	−1.427
LuLEA32	Lus10000125	LEA_5	1	4,694,779	4,694,943	165	54	5909.46	5.32	−1.339
LuLEA33	Lus10029709	LEA_6	5	1,264,925	1,265,191	267	88	9269.16	5.09	−1.016
LuLEA34	Lus10042745	LEA_6	10	13,884,863	13,885,135	273	90	9526.45	5.13	−1.076
LuLEA35	Lus10017977	Dehydrin	14	16,763,128	16,764,061	934	217	22,733.12	6.1	−0.812
LuLEA36	Lus10003340	Dehydrin	14	4,685,613	4,686,676	1064	178	19,706.95	5.3	−1.212
LuLEA37	Lus10041969	Dehydrin	11	2,850,938	2,851,697	760	218	23,017.47	5.94	−0.750
LuLEA38	Lus10005652	Dehydrin	8	690,871	691,579	709	201	22,112.42	5.85	−1.309
LuLEA39	Lus10021827	Dehydrin	2	19,108,891	19,109,681	791	225	24,978.48	5.21	−1.381
LuLEA40	Lus10034568	Dehydrin	13	13,731,116	13,731,893	778	229	25,306.88	5.48	−1.365
LuLEA41	Lus10014280	Dehydrin	2	3,310,261	3,310,809	549	154	16,240.58	9.07	−1.207
LuLEA42	Lus10025983	Dehydrin	13	3,173,711	3,174,236	526	146	15,485.83	9.05	−1.222
LuLEA43	Lus10021240	Dehydrin	6	15,878,896	15,879,638	743	215	23,562.05	6.03	−1.351
LuLEA44	Lus10022643	Dehydrin	1	10,316,073	10,317,141	1069	179	19,861.18	5.47	−1.226
LuLEA45	Lus10015948	SMP	14	10,954,195	10,955,467	1273	204	21,225.88	6.83	−0.311
LuLEA46	Lus10019001	SMP	3	15,348,416	15,350,123	1708	261	26,890.83	4.92	−0.336
LuLEA47	Lus10010553	SMP	8	21,761,528	21,762,395	868	259	26,464.46	4.87	−0.248
LuLEA48	Lus10022058	SMP	9	16,956,079	16,956,709	631	122	12,369.7	4.69	−0.234
LuLEA49	Lus10006121	SMP	8	13,816,075	13,816,961	887	264	27,082.12	4.76	−0.247
LuLEA50	Lus10042604	SMP	9	16,956,079	16,957,417	1339	263	26,484.21	4.68	−0.316

According to the chromosomal locations of *LuLEA* genes noted in the NCBI database, we generated distribution profiles of 49 *LuLEA* genes for analysis (Supplemental Fig. 1). It was clear that chromosome1 had the largest number of *LuLEA* genes up to 14. Other chromosomes had fewer than 6 *LuLEA* genes. Except for the LuLEA_6 and LuSMP subfamilies, other subfamilies had 1 to 3 members located on chromosome1. We further mapped the other 14 chromosomes of flax and found they had one to six *LuLEA* genes. For example, chr4, chr5 and chr7 had only one *LEA* gene on each chromosome, while chr11, chr12, chr13 and chr15 had two *LEA* genes on each chromosome (Supplementary Fig. 1).

The physicochemical parameters of these 50 *LuLEA* genes were attained using ExPASy. With the exception of one gene fragment (*LuLEA17*) being 14,123 bp in length, the *LuLEA* gene fragments ranged from 165 bp (*LuLEA32*) to 1708 bp (*LuLEA46*). A majority of the members encoded less than 300 amino acids. Members in the same subgroup displayed similar features. For example, as the members of the LuLEA_4 group, both *LuLEA26* (398 aa) and *LuLEA27* (497 aa) encoded remarkably large numbers of amino acids, while members of the LuLEA_3 group (*LuLEA18*–*LuLEA25*) encoded relatively small numbers of amino acids ranging from 81 to 109 aa. Likewise, molecular masses had the same pattern as amino acid numbers. Approximately two-thirds of the LuLEA proteins had relatively low isoelectric points (pI < 7), which consisted of all proteins in the LuLEA_2, LuLEA_4, LuLEA_5, LuLEA_6, and LuSMP subfamilies and some proteins in the dehydrin subfamily. The remaining proteins, in particular, both LuLEA_1 and LuLEA_3 subfamilies had pI > 7, meanwhile, LuLEA41 and LuLEA42 in dehydrin subfamily also had pI > 7. One-tenth of LuLEA proteins had relatively high values of grand average of hydropathicity (GRAVY > 0), and all of these proteins belonged to the LuLEA_2 subfamily. The data indicated that most LuLEA proteins were hydrophilic, especially those in LuLEA_5, while those in LuLEA_2 were determined as the most hydrophobic, which is consistent with the idea of atypical. Predictions of subcellular location showed that nearly 80% of LuLEA proteins were located in the nucleus. Only the LuLEA6 protein belonging to the LuLEA_1 subfamily was predicted to have a high possibility of being located in the plasma membrane. Interestingly, half of the LuLEA_3 proteins may be found in the chloroplast, and the other half of these members may be found in the mitochondrion. Moreover, LuLEA11 protein was also predicted to be in the chloroplast, and all of LuLEA_6 proteins with LuLEA2 were likely distributed in extracellular spaces (Supplementary Table 1).

### Biological evolution and gene structure analysis of *LuLEA* genes

**Fig. 1:**
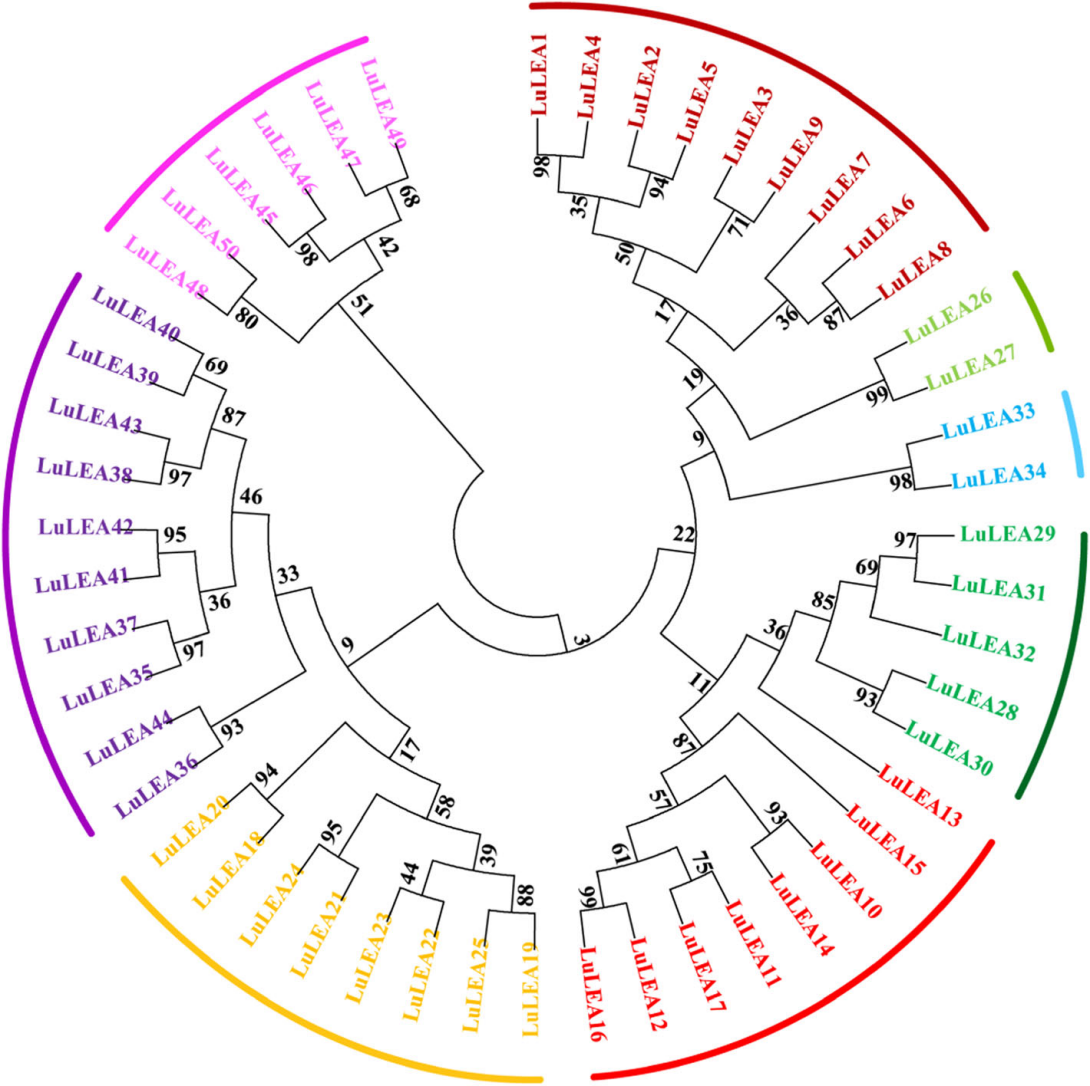
Phylogenetic analysis of the *LuLEA* genes in flax. *LuLEA* gene families are grouped by different colors. The unrooted tree was generated with the full-length amino acid sequences of the 50 LuLEA proteins using ClustalW in MEGA6 software

To investigate the homology and similarity for the identified *LuLEA* genes, an unrooted phylogenetic tree was constructed based on the alignment of all LuLEA protein sequences (Fig. 1). These genes divided into eight main clades, and the eight subfamilies of LuLEA protein sequences shared very low similarity. In contrast, high similarity was observed between a considerable number of proteins paired at the end of the branches, such as LuLEA1 and LuLEA4, LuLEA12 and LuLEA16, LuLEA35 and LuLEA37, which suggests there were still quite a few LuLEA members belonging to the same subfamily existing fair homology.

**Fig. 2:**
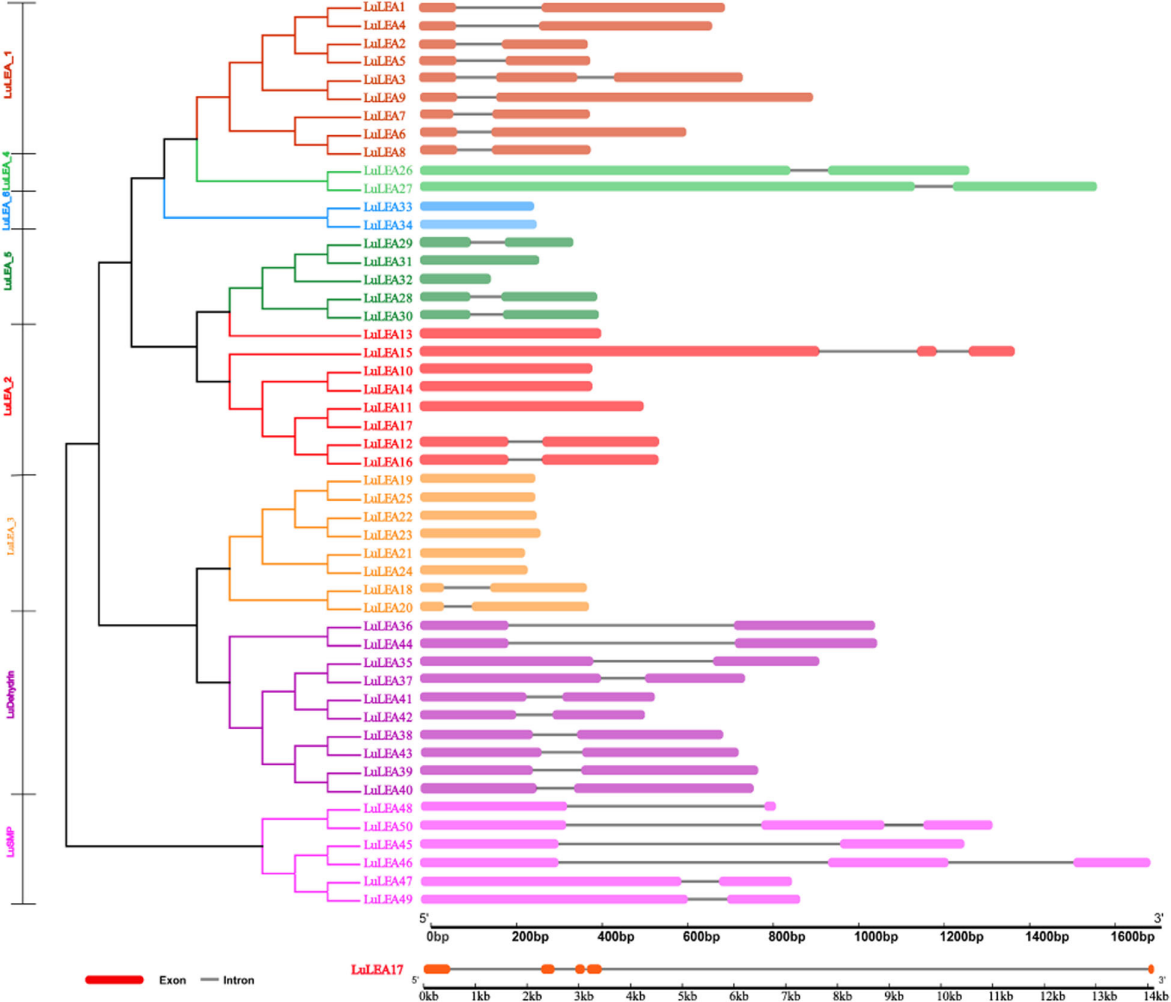
The distribution of exons and introns in *LuLEA* genes. Colored boxes represent exons, and grey lines indicate introns. The LEA subfamilies are distinguished by color. The lengths and positions of exons and introns in *LuLEA* genes are indicated by the scales at the bottom

The distribution of exons and introns in the genetic sequences of the *LuLEA* genes are shown in Fig. 2. Approximately all genes longer than 400 bp contained both exons and introns. Those genes lacking introns were found in three subfamilies: LuLEA_2, LuLEA_3 and LuLEA_4. Most genes having introns had only one intron. Also worth noting is that *LuLEA17*, which grouped into the LuLEA_2 subfamily, had the longest length than any other gene, up to 14 kb, and it also had the largest number of introns (4) and exons (5). Furthermore, the longest intron in *LuLEA17* was up to 10 kb in length.

**Fig. 3:**
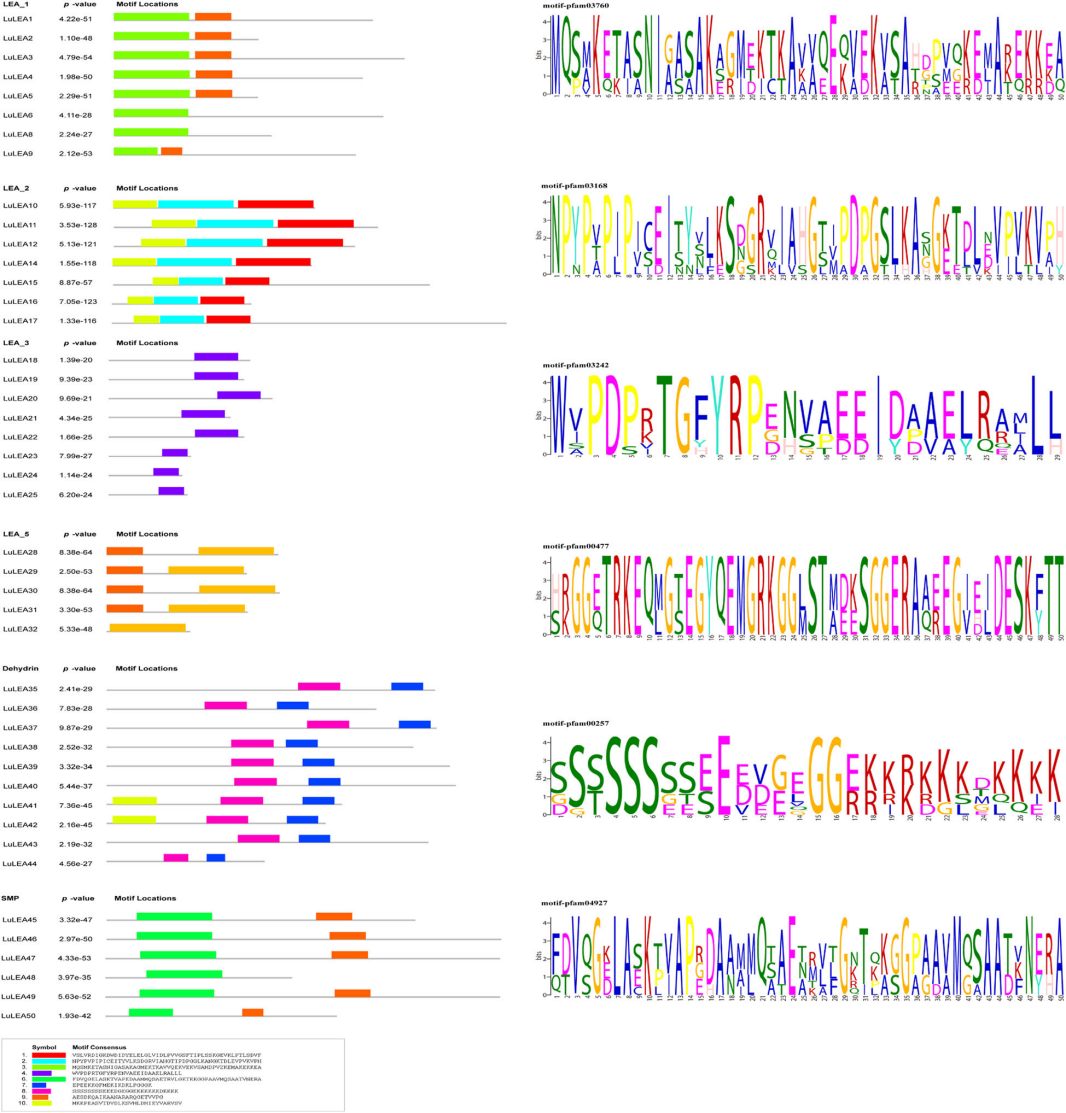
The distribution of motifs in *LuLEA* genes and the conserved amino acids in each subfamily exhibited by WebLogo plot. Different motifs are distinguished by different colored boxes. The maximum number of motifs in each sequence was set to 10. The representative motif of each subfamily is shown on the right

In addition to a gene sequence structure analysis, the distribution of motifs of each protein sequence was analyzed (Fig. 3). A total of 50 LuLEA protein sequences were submitted to MEME tool to determine the characters of the motifs. In general, one to three motifs were found for each subfamily and the motifs differed greatly among subfamilies (Fig. 3). The LEA_4 and LEA_6 subfamilies had too few motifs in common with those of the other subfamilies and thus were not shown in the results. Nevertheless, much similarity was observed in the numbers and types of members with in the same subfamily, which reflects the credibility of the phylogenetic analysis. Remarkably, the dehydrin subfamily had plenty of conserved hydrophilic amino acids, such as G (Glycine) and K (Lysine), which implies a subfamily trait of hydropathy.

### Gene expression pattern analysis of *LuLEA* genes during seed developing stages

**Fig. 4:**
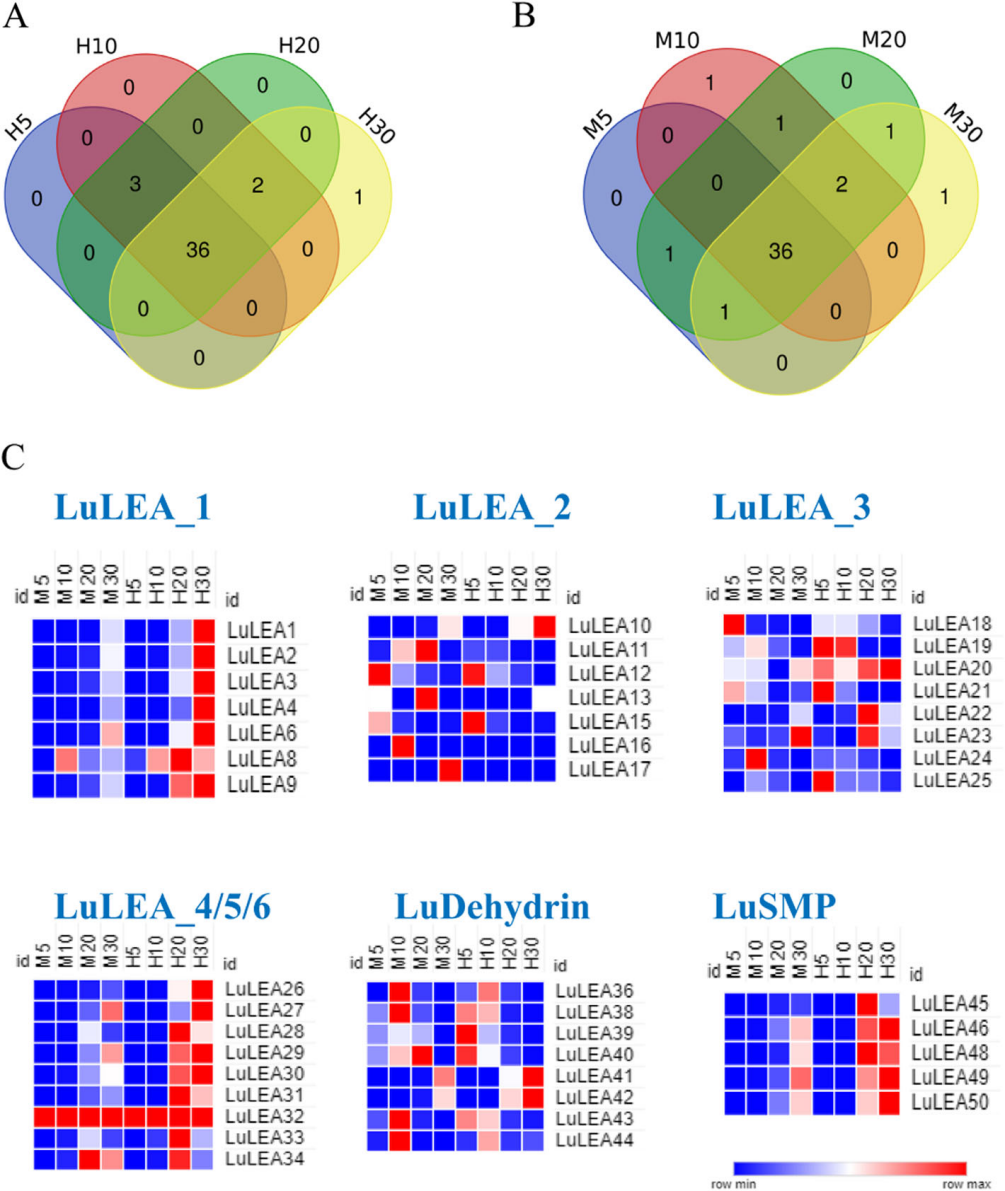
Expression profiles of *LuLEA* gene families in flax seed development. **a** Venn diagram of shared and non-shared numbers of genes of the cultivar Heiya No.14 expressed at 5, 10, 20, and 30 days after pollination; **b** similar to **a** but of the cultivar Macbeth; **c** comparison of *LuLEA* gene expression levels in every subfamily during seed maturation. Shades of blue color represent lower expression levels, and shades of red color represent higher levels

The expression patterns showed that nearly all of the *LuLEA* genes expressed throughout all stages of seed development for both of our flax cultivars, Heiya No.14 and Macbeth. In Heiya No.14, a total of 42 *LuLEA* genes expressed during all stages, and 44 *LuLEA* genes expressed in Macbeth. In comparing the commonly expressed genes between these two cultivars, we found that 36 *LuLEA* genes expressed over 5 days, 10 days, 20 days 30 days after pollination (DAP); one gene express at the 30th day of seed development. Additionally, there was also only one gene that expressed at the 10th day in Macbeth but not in Heiya No.14, which signifies another difference between the two flax cultivars (Fig. 4a-b).

To confirm the observed variation in expression patterns among members in the LEA subfamilies, heat maps were produced for individual subfamilies. The trends of most *LuLEA* gene expression levels were consistent between Heiya No.14 and Macbeth. Some *LuLEA* genes, such as *LuLEA1*, *LuLEA2* and *LuLEA41*, tended to highly express at late stages of seed development. On the contrary, expression of a few *LuLEA* genes, such as *LuLEA15*, *LuLEA38*, and *LuLEA43*, decreased from early to late developmental stages. Genes in the five main *LuLEA* subfamilies, LuLEA_1, LuLEA_4–LuLEA_6, and LuSMP, displayed similar expression patterns. An exception was observed in *LuLEA32* where this gene highly expressed throughout our four sampling periods. The rest of the genes in the five subfamilies exhibited increased expression largely at days 20 and 30 (Fig. 4c).

### *LuLEA1* is responsible for seed development and fatty acid metabolism

**Fig. 5:**
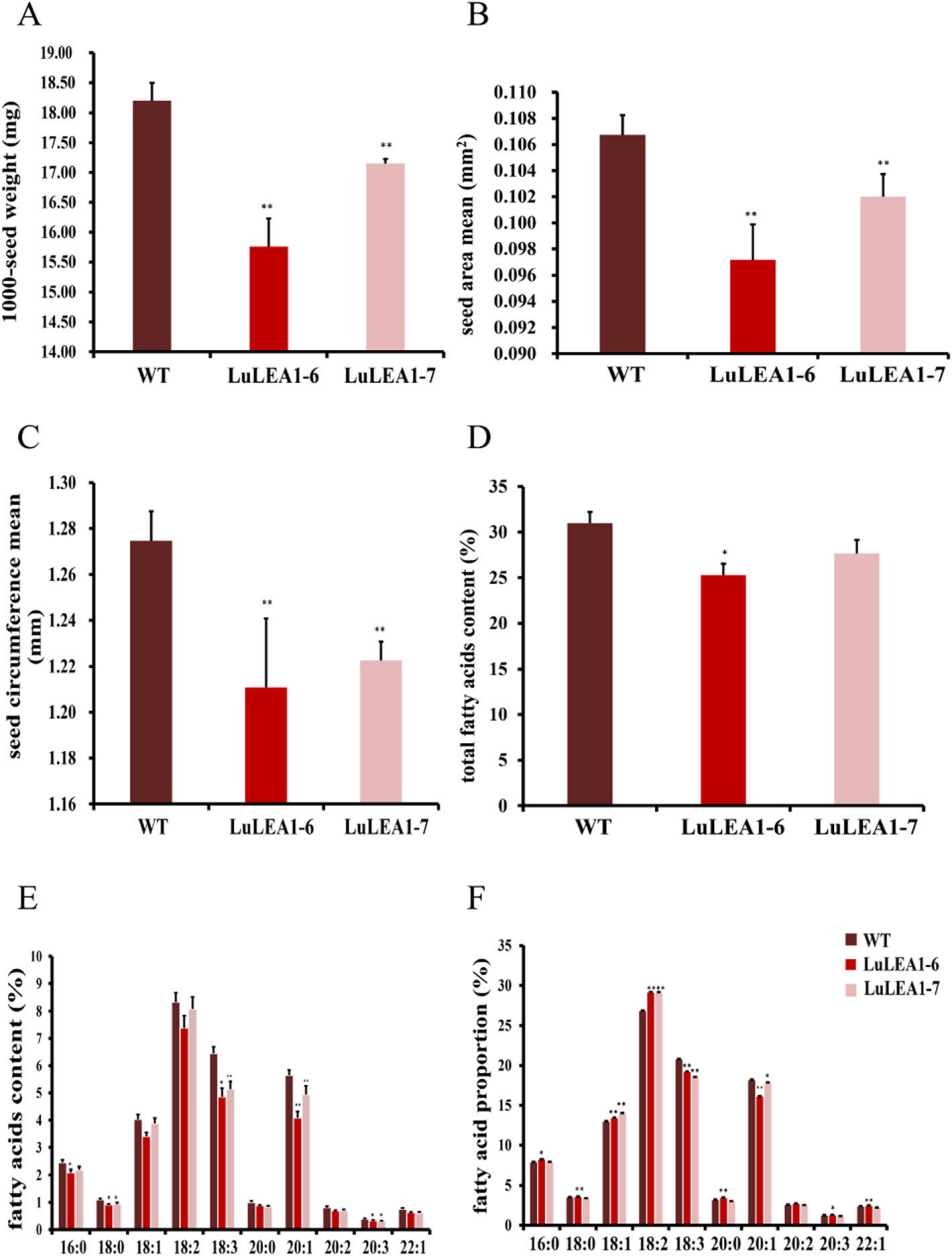
Effect of *LuLEA1* on seed size and fatty acid content in transgenic *Arabidopsis* lines. **a** The 1000-seed weights of transgenic LuLEA1 lines and wild type grown under the “normal” condition; **b** the average area of transgenic and WT seeds; **c** the average circumference of transgenic and WT seeds. **d** Total fatty acid contents of overexpression lines and wild type grown under the “normal” condition; **e** 10 fatty acid components in overexpression lines and wild type grown under the “normal” condition; **f** proportions of each fatty acid component. The data represent means and standard deviations (STD) of at least three replicates. Statistically significant differences were determined by two-tailed paired Student’s t-tests comparing transgenic data with WT data of plants grown under the same condition. * *p* < 0.05; ** *p* < 0.01

Two independent overexpression lines, named LuLEA1–6 and LuLEA1–7, were generated and analyzed. Compared to those of the WT plants, both of the two overexpression lines had significantly lower values of the measured seed traits, seed weight, area and circumference. The results indicate that *LuLEA1* may play a role in regulating seed size (Fig. 5a-c).

To further explore whether *LuLEA1* functions in controlling fatty acid metabolism during seed development, fatty acid content in the transgenic *Arabidopsis* lines were determined by GC-MS (Gas Chromatography-Mass Spectrometer). Total average fatty acid contents of the two overexpression lines were less than that of WT, and LuLEA1–6 was significantly reduced. Meanwhile, most contents of the different types of fatty acids of the transgenic lines were lower than those of WT, and the contents of C18:0, C18:3, C20:1 and C20:3 were significantly reduced. In addition, we found that the proportion of each fatty acid differed, too. The proportions of C18:1 and C18:2 in transgenic lines were markedly higher, while the proportions of C18:3 and C20:1 were lower than WT. These results suggest that *LuLEA1* may block the process of transformations of C18:1 and C18:2 into C18:3 and C20:1 (Figs. 5d-f).

## Discussion

The LEA gene family is a large and complicated family, having many members that belong to different subfamilies. Genes in the LEA family have been identified in many crops, such as rice, *A. thaliana* and wheat. Besides in plants, this family of genes has been reported in both animals and microorganisms. However, characterization and identification of the LEA protein family in flax has never been reported. In this research, 50 *LEA* genes were identified in the flax genome, nearly equal to the 51 *LEA* genes found in *A. thaliana*. Given that flax is diploid (2n = 30) and the number of *LuLEA* is close to that of *A. thaliana*, whole genome duplication events of *LEA* genes occurring in flax was doubtful and supported by many findings of the evolutionary conservation of *LEA* genes [22, 29]. Thus, it is easy to deduce that *LEA* must play a crucial role in the development of organisms.

The 50 *LEA* genes in flax were divided into eight subfamilies. Among the subfamilies, the dehydrin subfamily has the greatest number of genes, 10, in the LuLEA family, while the LuLEA_6 subfamily has the least with 2. The distributions of the *LEA_6* and *dehydrin* genes in flax are similar to those in *A. thaliana*. From multiple plant species comparisons, although some are largely occupied with LEA_4 subfamily or LEA_2 subfamilies, such as *A. thaliana*, *B. napus*, cotton (*Gossypium hirsutum*), tea (*Camellia sinensis*), dehydrin subfamily tends to share considerably part, which means dehydrin is relatively conserved and likely to provide more stable protection for cells during the evolution. Evidence shows that the LEA_6 subfamily is not found in algal and rice genomes [6, 22], which suggests LEA_6 was extended from other ancient LEA genes, and probably makes contribution to struggling with the water loss.

Based on our results, five *LuLEA_2* genes likely encode hydrophobic proteins, while the others are hydrophilic proteins. This result is consistent with the results of past research on *A. thaliana*, *Populus trichocarpa*, and *Solanum tuberosum*. *LEA_2* genes are thought to be heterologous to other subfamilies of *LEA* genes, which may explain the unique structures, atypical characters and even novel functions reported of members in the LEA_2 subfamily [3].

In flax, *LEA_2* genes may only be present in chloroplasts and mitochondria, which indicates that *LEA_2* may function in protecting proteins in these particular cellular organelles. There were also some LuLEA proteins in the nucleus and cytoplasm as well as cytoplasmic membranes. These results indicate that LEA proteins are widely distributed within cells, so these proteins having an important role such as protection of cellular compartments during stressful conditions is not without support. Moreover, most of our identified LuLEA proteins are hydrophilic according to their GRAVY values, which is quite similar to characterizations determined of LEA proteins in other higher plants [5, 9, 29]. Many studies have shown that the trait of high hydrophilicity is attributable to the presence of IDPs in LEA proteins, and high hydrophilicity facilitates their potential functions as protein and membrane protectants and molecular chaperones to ensure cellular survival in a variety of adverse environments.

The map of gene structures containing introns and exons clearly show a large number of *LuLEA* genes possessed less than 2 introns and relatively short gene lengths. One previous study showed that genes associated with stress response have few introns [37], which is supported by our results. Reports of many *LEA* genes with few introns in other plant species confirm this as well. In *B. napus*, 16/108 *BnLEA* genes have no introns, and the subfamily BnLEA_6 has five members that each have only one intron [29]. In wheat, 62% of its *LEA* genes have no introns [32]. In *A. thaliana*, 66.7% of its *LEA* genes contain only one intron [5]. In addition, similar conclusions of low intron numbers have been reported in other genes known to be involved in stress responses. For example, most *StHsp20* genes (89.6%) with no or only one intron were demonstrated to respond to multiple abiotic stresses [38]. In another example, a high percentage (83.9%) of the zinc finger homeodomain genes that encode transcription factors involved in plant development and abiotic stress response in *B. napus* lack introns [39]. From the perspective of biomolecular activities, introns will be spliced out of the final sequence after transcription. Reduced introns of genes are benefit for the faster process from transcription to expression, which is convenient for cell to make a reaction to abiotic stresses and decrease the cost for transcription [40].

Different LuLEA subfamilies have various motif distributions. Proteins belonging to the same subfamily have similar numbers and types of motifs, which is illustrated by our phylogenetic tree. Maybe these characters imply the reasons for various functions of LEA proteins.

In most cases, gene expression analysis can help reveal important functions of target genes. According to the expression pattern of *LuLEA* genes at 5, 10, 20, and 30 DAP, only six *LuLEA* genes lacked expression during linseed maturation, while the other genes expressed throughout the entire process. These observations suggest that these genes play vital roles in the seed maturation process. Additionally, expression of members in several subfamilies, such as LuLEA_1, LuLEA_4 ~ LuLEA_6 and LuSMP, accumulated in abundance in late seed maturation, which is consistent with the reported data of previous studies [18, 19, 26]. These *LuLEA* genes were speculated to play an important part in seed maturation and desiccation. Meanwhile, some *LuLEA* genes, such as *LuLEA_3* and *Ludehydrin* appeared no clearly regularity, which may explain the diversity of potential functions of LEA proteins and the correlations to the various structures.

Past studies have shown that *LEA* genes participate in the regulatory network of seed development [18], thus we investigated the phenotypes of seeds produced from *LuLEA1*-overexpressing transgenic *Arabidopsis*. The traits of seed weight, area and circumference were all reduced. Furthermore, fatty acid contents in seeds also declined. Based on those results, we conclude that the LEA_1 subfamily of genes negatively regulate seed size and fatty acid contents. Interestingly, Liang et al. [27] showed the opposite result: overexpression of a gene belonging to the LEA_4 subfamily, *BnLEA3*, could increase seed size and seed oil content in *Arabidopsis*. However, there is no evidence indicating the direct involvement of *LEA* genes in the regulatory mechanism of seed size and oil synthesis. Based on existing findings, LEA proteins are regulated by transcription factors ABI3, ABI4, ABI5 [18], and these factors have also been shown to affect seed size and lipid biosynthesis [23, 41, 42]. Thus, LEA proteins likely have a feedback relationship with these transcription factors, and different LEA families may have contrasting functions conferred by their different subfamilies to maintain a balance among functions in collectively protecting a plant.

## Conclusions

In this research, a total of 50 *LEA* genes were identified in the flax genome, and they were divided into eight subfamilies based on their conserved domains. Genes from the same subfamily had similar structures, which is also supported by the results of phylogenetic analysis. All *LuLEA* genes were distributed on each chromosome. The overexpression of *LuLEA1* in *Arabidopsis* decreased the traits of seed weight and size, as well as fatty acid contents.

## Methods

### Identification of *LEA* gene family members in the flax genome

Fifty-one *LEA* gene sequences of *A. thaliana* were retrieved from the database TAIR (The Arabidopsis Information Resource, https://www.arabidopsis.org/), and then they were blasted using protein sequences of flax acquired from the genome database Phytozome (https://phytozome.jgi.doe.gov/pz/portal.html#!info?alias=Org_Lusitatissimum). We also used the Pfam database (https://pfam.xfam.org/search) and HMMER to search for the genes with the conserved LEA domain [43]. Combining BLAST with HMMER, the initial candidate LEA genes of flax were obtained after filtering the mismatched or redundant genes. Three website tools, CDD (Conserved Domain Database, https://www.ncbi.nlm.nih.gov/cdd/), Pfam and SMART (https://smart.embl-heidelberg.de/smart/set_mode.cgi?NORMAL=1) were used to confirm and ensure all candidate genes contained the LEA family domain. The final filtered genes were assigned new names in numbered order.

The number of amino acids and gene lengths were obtained through the Phytozome web portal (https://phytozome.jgi.doe.gov/pz/portal.html), and chromosome locations of the *LuLEA* genes were obtained from the NCBI database (National Center for Biotechnology Information, https://www.ncbi.nlm.nih.gov/). The physicochemical parameters, composed of molecular weight (kDa), GRAVY (grand average of hydropathy) and pI (isoelectric point), of each LuLEA protein were calculated by ExPASy (www.expasy.org/tools/). Subcellular location prediction was conducted using the BUSCA annotation system (https://busca.biocomp.unibo.it/).

### Phylogenetic and sequence feature analysis of LuLEA family members

Multiple sequence alignment of 50 LuLEA protein sequences was performed using ClustalW [44], and these results were used to construct a phylogenetic tree with the MEGA7 software [45]. The method of maximum likelihood was adopted to construct the tree, and it had 1000 bootstrap replicates. To understand the structural features of *LuLEA* genes, the genetic sequences containing exons and introns were examined, and the distributions of motifs on each protein sequence were determined. Owing to the variation between each sequence, the maximum value of motif for each gene was set as 10. In the gene structure analysis of *LuLEA* genes, which was limited to the annotation of flax, UTRs (untranslated region) could not be displayed. The distribution of intron and exon fragments on each *LuLEA* gene were visualized by a diagram with the help of the Gene Structure Display Server (https://gsds.cbi.pku.edu.cn/). The relative locations of conserved amino acid motifs encoded by *LuLEA* family genes were determined using Multiple Expectation Maximization for Motif Elicitation tool (https://alternate.meme-suite.org/). The chromosomal locations of *LuLEA* genes were derived from the positional information available in the NCBI website. The distribution of LuLEA family members on the chromosomes were visualized using MG2C (https://mg2c.iask.in/mg2c_v2.0/).

### RNA extraction and RNA-seq of developing seed samples

The flax cultivars Heiya No. 14 [46] and Macbeth were used as the plant materials for sample collection and RNA isolation. Heiya No.14 was bred for the purpose of better quality and high yields of fiber flax, and its seed oil content makes up about 25% of seed weight. Macbeth is an oilseed flax that produces about 40% seed oil content as well as large seed sizes. Plants were grown in a greenhouse under “normal” growth conditions of 24 °C and a 16 h daylight/8 h dark cycle. After plants reproduced, the siliques were collected at 5 days (DAP5), 10 days (DAP10), 20 days (DAP20), and 30 days after pollination (DAP30) and immediately frozen in liquid nitrogen before RNA isolation. Two replicates were prepared for the construction of a sequencing library per sample. Total RNA was isolated using TRIzol reagent (Invitrogen, 15,596–026), according to the manufacturer’s instructions. Then cDNA libraries were constructed and subsequently inspected. Based on sequencing by synthesis technology, the Illumina HiSeq 2500 platform was used to perform cDNA library sequencing and acquire a large amount of high-quality data.

### Gene expression pattern analysis for *LuLEA* gene families with RNA-seq data

We used RNA-seq data to analyze the gene expression patterns of *LuLEA* genes. After filtering the sequenced raw data, the clean data were mapped to the flax reference genome (https://phytozome.jgi.doe.gov/pz/portal.html). Then, the FPKM (Fragments per Kilobase of Exon per Million Fragments Mapped) method [47] was applied to calculate gene expression levels based on the number of reads mapped to the reference sequence. A heatmap of gene expression profiles of all *LuLEA* genes was constructed using Mev 4.0 software [48] with Pearson’s correction and complete linkage clustering. The raw data have been submitted to the NCBI database with the GEO number GSE130378.

### Vector construction, gene transformation, and phenotypic screening of transgenic plants

In order to test how *LuLEA* genes may affect plant development, we selected one *LuLEA* gene with high expression during late seed maturation for use in the genetic transformation of *A. thaliana*. The selected gene, *LuLEA1*, exhibited a level of expression at 30 DAP that was up to 10,000-fold that of the level at 5 DAP based on the RNA-Seq data. The RNA-Seq raw data is available in the NCBI database with the GEO number GSE130378 (https://www.ncbi.nlm.nih.gov/geo/query/acc.cgi?acc=GSE130378). The full-length CDS of *LuLEA1* was cloned into the CaMV 35S-Red vector. The plasmids were double digested with the restriction endonucleases *Xma*I and *EcoR*I and then ligated with the specific gene transcript fragment so that the gene expression of the target gene was under the control of the CaMV 35S promoter. The construct was transformed into *Agrobacterium tumefaciens* strain EHA105 using the freeze–thaw method. *Arabidopsis* Col-0 plants were then transformed using the floral dip method [49]. Untransformed *Arabidopsis* plants were used as WT controls. All plants were maintained in a greenhouse under standard conditions (24°Cday/18 °C night and 16 h light/8 h dark).

Transgenic plants were screened and cultivated to the T3 generation. Then the seeds were harvested, the size and weight of which were determined by a crop scanning test system (Wanshen SC-G, China) [27] and the Seed Count image analysis system [50]. And the fatty acid compositions in seed samples were quantified by gas chromatography mass spectrometry (GC-MS) [50].

## Supplementary Information

**Additional file 1: Supplementary Figure 1 The positions of** ***LuLEA*** **genes on chromosomes.** Each box represents a chromosome, where the *LuLEA* genes are mapped with the slim bar. The genes in the same subfamily are marked by identical coloring. The scale to the left of the chromosome is in millions of bases (Mb).

**Additional file 2: Supplementary Table 1** Subcellular localization prediction of all the 50 *LuLEA* genes.

## Data Availability

The raw RNA-seq data of cultivars Macbeth and Heiya No.14 of *Linum usitatissimum* L. obtained at different developmental stages of seeds are available in the NCBI database under the GEO number GSE130378 (https://www.ncbi.nlm.nih.gov/geo/query/acc.cgi?acc=GSE130378). All data generated or analyzed during this study are included in this published article and its supplementary information files. The datasets used and analyzed for the current study are available from the corresponding author upon reasonable request.

## Abbreviations

LEA: Late embryogenesis abundant protein
SMP: Seed maturation protein
ALA: Alpha-linolenic acid
IDPs: Intrinsically disordered proteins
PAL: Palmitic acid
STE: Stearic acid
OLE: Oleic acid
LIO: Linoleic acid
LIN: Linolenic acid
DAP: Day after pollination
GC-MS: Gas Chromatography-Mass Spectrometer
