# Supplementary figures and images for: Genome-wide identification and functional characterization of LEA genes during seed development process in linseed flax (Linum usitatissimum L.)
Source: BMC Plant Biol. 2021 Apr 21;21:193. doi: 10.1186/s12870-021-02972-0 (PMC8059249; doi:10.1186/s12870-021-02972-0)

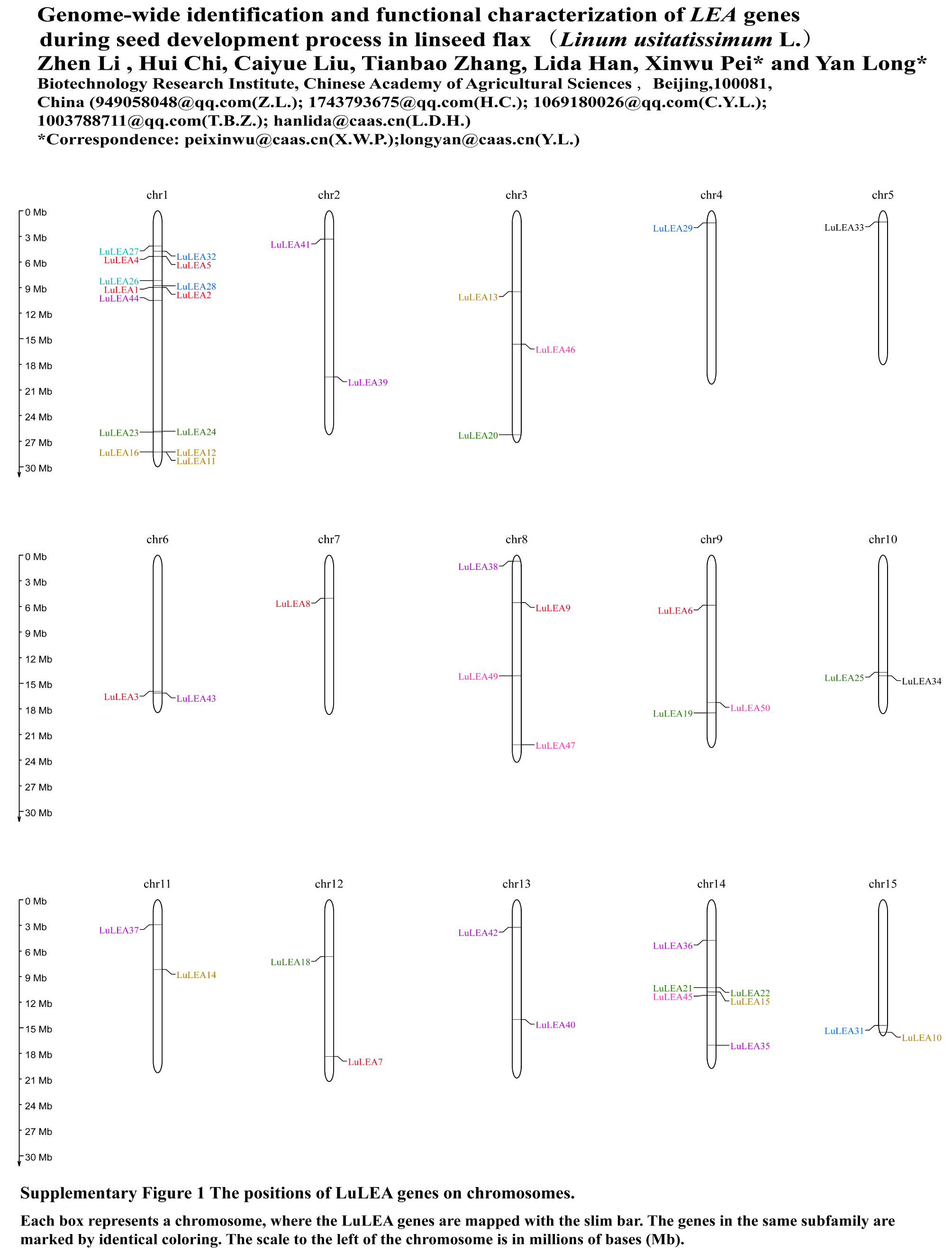

Supplement: Supplementary file 1 — Additional file 1: Supplementary Figure 1 The positions of LuLEA genes on chromosomes. Each box represents a chromosome, where the LuLEA genes are mapped with the slim bar. The genes in the same subfamily are marked by identical coloring. The scale to the left of the chromosome is in millions of bases (Mb). [file 12870_2021_2972_MOESM1_ESM.tif]
